# A systematic review of effectiveness of BNT162b2 mRNA and ChAdOx1 adenoviral vector COVID-19 vaccines in the general population

**DOI:** 10.1186/s42269-021-00607-w

**Published:** 2021-08-24

**Authors:** Chinonyerem O. Iheanacho, Uchenna I. H. Eze, Emmanuel A. Adida

**Affiliations:** 1 Department of Clinical Pharmacy and Public Health, Faculty of Pharmacy, University of Calabar, Calabar, PMB 1115 Nigeria; 2 Department of Clinical Pharmacy and Biopharmarcy, Faculty of Pharmacy, Olabisi Onabanjo University, Sagamu, Nigeria

**Keywords:** COVID-19, SARS-CoV-2, ChAdOx1 vaccine, BNT162b2 mRNA vaccine, Effectiveness, Adenovirus vector-based vaccine

## Abstract

**Background:**

High effectiveness of COVID-19 vaccines is essential for the pandemic control. This study systematically reviewed available evidence on effectiveness of ChAdOx1 and BNT162b2 vaccines in the general population, for improved vaccine policies and strategies.

**Main body of the abstract:**

Using several keywords, a search of Scopus, PubMed, Google scholar and Hinari databases was conducted from December 1, 2020 to June 9, 2021. Eligible studies comprising original studies reporting effectiveness of the vaccines, were included following PRISMA guidelines. Individual studies were assessed for quality using National Heart, Lung and Blood Institute quality assessment tool. A total of 1766 titles were retrieved and 11 were included, out of which only 5 were peer-reviewed. Although data availability was limited, studies suggest equivalent effectiveness of BNT162b2 and ChAdOx1 COVID-19 vaccine against SARS-CoV-2 infection and COVID-19 related morbidity and mortality. Vaccine effectiveness increased steadily to about 35 days, with an enhanced effectiveness following the second dose.

**Short conclusion:**

BNT162 and ChAdOx1 vaccines were associated with equivalent and high effectiveness which increased with time and a second dose in the general population. This encourages continued practice of other preventive measures, particularly during the first week of vaccination, and reinforces the need for a second dose.

## Background

The origination of Severe Acute Respiratory Syndrome Coronavirus 2 (SARS-CoV-2) in late 2019 has been characterised with several forms of loss of social and economic activities (Nicola et al. 2020). Being a global outbreak, coronavirus disease 2019 (COVID-19) has resulted in several morbidities and mortalities across all continents (Sanyaolu et al. 2020). As of June 17, 2021, a total of 177,108,695 cases of COVID-19 had been confirmed globally, with a total of 3,840,223 deaths (World Health Organisation 2020a). The rapid spread of SARS-CoV-2 infection and its consequences across the globe necessitated a corresponding wave of vaccine development (Forni and Mantovani 2021), and the emergency use authorisation of some vaccines in various countries (World Health Organisation 2020b). The messenger RNA vaccines and the Chimpanzee adenovirus vector vaccines appear to be most widely used in several countries. Being critical for effective control of the pandemic, COVID-19 vaccine safety and effectiveness is a vital focal point. Reports of interim analysis of a clinical trial associated ChAdOx1 vaccine with acceptable safety and efficacy against symptomatic COVID-19 (Voysey et al. 2021). Effectiveness of 95% was also reported of BNT162b2 in preventing COVID-19 after a two-dose regimen (Polack et al. 2020), and a total of 2,378,482,776 vaccine doses have been administered as at June 17, 2021 (World Health Organisation 2020a).

Meanwhile, clinical trials of the safety and effectiveness of COVID-19 vaccine have had low inclusion of vulnerable groups particularly older persons, who become recipients after vaccine roll-outs (Helfand 2020; Cooper et al. 2021). It has been argued that exclusion of vulnerable groups in clinical trials does not allow availability of data that enables adequate understanding of the influence of intervention in the groups (Welch et al. 2015). Hence, pharmacovigilance of the rolled out vaccines is a critical need for evaluating its effectiveness across all groups including older persons. This post marketing surveillance of new drugs is a critical aspect of evaluating medicine safety and effectiveness, particularly in groups that are usually considered ineligible for inclusion in Phase 2 and 3 trials (Raj et al. 2019). Real world effectiveness of available vaccines against a range of SARS-CoV-2 outcomes is crucial for the determination of health impacts of the vaccines.

Evaluating dose-dependent vaccine effectiveness is also increasingly important, particularly in light of extended dosing intervals that have been implemented in order to maximise vaccine coverage across high-risk groups (https://www.gov.uk/government/news/statement-from-the-uk-chief-medical-officers-on-the-prioritisation-of-first-doses-of-covid-19-vaccines). It is also essential to evaluate the effectiveness of the first vaccine dose at various time intervals, including less than 7 days, after 8–12 weeks and above, as well as several days after second dose, and to evaluate the long-term impact of vaccination on SARS-CoV-2 infection, transmission and mortality. Effectiveness describes the ability of the vaccines to successfully produce intended results. This review systematically assessed evidence of “real world” effectiveness of BNT162b2 and ChAdOx1 vaccines in the general populations. It measured the vaccines dose-dependent effectiveness in preventing SARS-CoV-2 infection, COVID-19 related morbidity and mortality, and interval between vaccine administration and observed effectiveness. It also assessed potential differences in effectiveness of BNT162b2 and ChAdOx1 vaccines in recipients. This will inform policy decisions regarding the ongoing need for the control of SARS-CoV-2 and COVID-19.

## Main text

### Study design

The review was performed in accordance with Preferred Reporting Items for Systematic Reviews and Meta-Analysis (PRISMA) guidelines 2009 (Moher et al. 2009). Systematic review of eligible articles was conducted from electronic databases of PubMed, Scopus, Hinari and Google scholar from December 1, 2020 to June 9, 2021.

### Search strategy

With the use of keywords in various combinations, authors conducted an independent search to reduce potential bias. The keywords were: COVID-19 vaccine, BNT162b2, mRNA, side effect, ChAdOx1, adenoviral vector vaccine, efficacy, and effectiveness.

### Inclusion and exclusion criteria

After a careful assessment of titles and abstracts of studies, only articles that reported studies on outcomes of the available COVID-19 vaccines were assessed for eligibility. Duplicates where eliminated and full text of papers were independently reviewed by the authors to determine quality of the articles. A random 10% of titles was also checked by an author to ensure relevant titles were not excluded.

Inclusion criteria.

Original articles that reported effectiveness of BTN162b2 COVID-19 vaccine.

Original articles that reported effectiveness of ChAdOx1 adenovirus vector vaccine.

Studies written in English language.

Articles were excluded if they were:

Reviews, correspondences, viewpoints and commentaries on BTN162b2 and ChAdOx1 vaccines.

Animal studies on BTN162b2 and ChAdOx1 vaccine trials.

Clinical trials of BTN162b2 and ChAdOx1vaccines.

### Outcome measures

Outcomes of interest were.

Documented PCR confirmed infection with SARS-CoV-2 infection after vaccination.

Documented symptomatic COVID-19 after vaccination after vaccination.

Death from COVID-19 after vaccination.

### Evaluation of the selected studies and method of risk of bias assessment

Critical evaluation of all included studies was done, and outcomes of interests were noted. Individual studies were assessed for limitations at the study level, and quality of included studies was assessed using the National Heart, Lung and Blood Institute (NHLBI) quality assessment tool (Study Quality Assessment Tools. https://www.nhlbi.nih.gov/).

### Data extraction and synthesis

Extracted data included study authors, study period, study design, study size, and results. Data were extracted independently by the authors to reduce risk of bias, and results were categorised into 4 sections.

### Search results

**Fig. 1:**
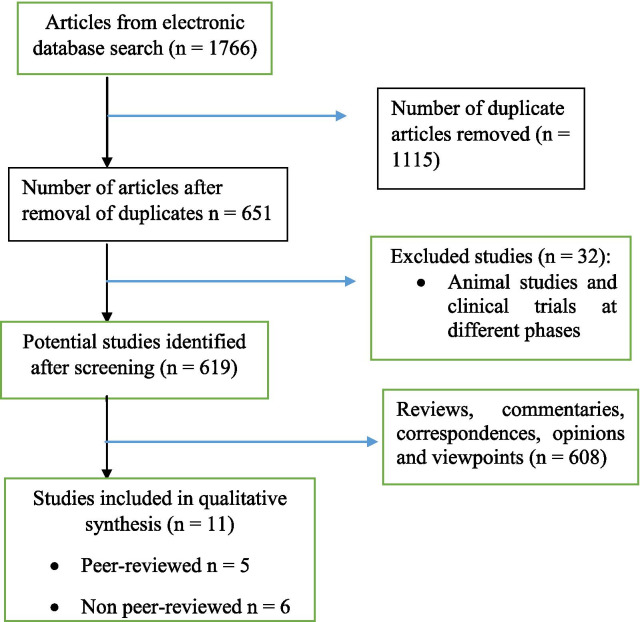
PRISMA flow diagram of study selection

A total of 1766 titles were retrieved, 619 studies were assessed after screening, and 11 studies met the inclusion criteria of our review. Study selection process is shown in the PRISMA flow diagram (Fig. 1).

### Characteristics of included studies

A total of 11 studies were included in the study and 5 were peer-reviewed, while 6 studies were not. (See Table 1). From the NHLBI quality assessment, most of the studies were of high quality. All the studies were observational, with 10 being retrospective studies.

**Table 1:** Characteristics of included studies showing effectiveness of BNT162b2 and ChAdOx1 COVID-19 vaccines

Study location	Study design	Sample size	Effectiveness of BNT162b2	Effectiveness of ChAdOx1	Study limitations
Israel Dangan et al. (2021)	Retrospective	596,618	Effectiveness increased with increase in time, and with receipt of second dose Effectiveness against symptomatic COVID-19 was 57% after 14–20 days and 66% after 21–27 days	–	Short follow-up after second dose, potential selection bias, risk of potential confounders, Potential exclusion of some eligible participants as a result of efforts to match
Israel Chodlick et al. (2021)	Retrospective	503,875 (≥ 16 years)	51% effectiveness after within 24 days of first dose, effectiveness increased with increase in time	–	Potential unreported vaccination among potential eligible participants, not peer-reviewed as at the time of this review
England Barnal et al. (2021a)	Retrospective	174,731 (≥ 70 years old)	Single dose was 80% effective at preventing hospitalisation, 85% effective at preventing COVID-19 related death	Single dose was 80% effective at preventing hospitalisation	Inherent limitations of observational study, potential confounders, yet to be peer-reviewed as at the time of this review
Denmark Moutsen-Helms et al. (2021)	Retrospective	39,040 persons of 77–90 years and 33 039 persons of 36–57 years old	Protective effect was absent in older persons after first dose. Vaccine effectiveness was higher in the younger population (90%) than in older persons (64%) after 7 days of second vaccine dose	–	Study was not peer-reviewed as at the time of this study, observed variation in vaccine effectiveness may have been influenced by the large variation in sample size of the groups and potential confounders
Scotland Vasileiou et al. (2021)	Retrospective	1,331 993 (Mean age of 65 years old)	First dose was associated with 91% reduced COVID-19—related hospitalisation at 28–34 days after vaccination	First dose was associated with 88% reduced COVID-19—related hospitalisation at 28–34 days after vaccination	Risk of potential confounders
England Bernal et al. (2021b)	Retrospective	48,096 (≥ 70 years old)	Associated 44% and 69% reduced risk of death with one and two respective doses of BNT162b2 vaccination among COVID-19 cases	Associated 55% reduced risk of death from COVID-19	Insufficient follow-up to ascertain effects of second dose of ChAdOx1 vaccine on risk of death. Yet to be peer-reviewed at the time of this study
England Shroti et al. (2021)	Retrospective	10,412 (older adults)	Reduction in SARS-CoV-2 infection after first and second dose	Reduction in SARS-CoV-2 infection after first and second dose	Study was yet to be peer-reviewed as at the time of this review. Risk of potential confounder bias may also be associated with the study
England Hall et al. 2021	Prospective	23,324 (median age = 46.1)	Associated with 72% effectiveness after 21 days of first dose, and 86% effectiveness seven days after second dose	–	Risk of potential confounders
United Kingdom Pritchard et al. (2021)	Retrospective	383,812 (≥ 16 years old)	Reduced SARS-CoV-2 infections ≥ 21 days after the first dose at 66% and 80% after second dose	Reduced SARS-CoV-2 infections ≥ 21 days after the first dose at 61% and 79% after second dose	Risk of potential confounders
Israel Haas et al. (2021)	Retrospective	(≥ 16 years old)	Associated with 95.3% preventing incidence of SARS-CoV-2 7 days after second dose, 97.2% and 96.7% against COVID-19 related hospitalisation and death respectively	–	Risk of potential confounders
England Bernal et al. (2021)	Retrospective	12,675	Dose effectiveness of BNT162b2 reduced from 93.4% with B.1.1.7 to 87.9% with B.1.6172.2	Associated effectiveness of two doses reduced 66.1% with B.1.1.7 to 59.8% with B.1.6172.2	Potential misclassification of cases and control may have been influenced by sensitivity and specificity of the PCR, paper was yet to be validated by peer-review as at the time of this review

### Effectiveness of BNT162b2 mRNA and ChAdOx1 adenoviral vector COVID-19 vaccines against SARS-CoV-2 infections and COVID-19 morbidity and mortality

A study of COVID-19 vaccine effectiveness in Israel showed that BNT162b2 mRNA vaccine was effective for a wide range of COVID-19-related outcomes. In the retrospective study, data of vaccinated persons were collected from December 20, 2020 to February 1, 2021 and matched with unvaccinated controls on a 1:1 ratio. The cohort was matched on variables associated with probability of vaccination and infection, severity of infection and socio-demographics. From a population of 3,159,136 of local healthcare register, vaccinated population of 596,618 were matched to an unvaccinated population of 596,618 from whom 86, 601 were re-matched to the vaccinated cohort after being vaccinated. Researchers in this study (Dagan et al. 2021) observed that the unvaccinated cohort was younger than the vaccinated group and had lower prevalence of diseases. This was probably a result of higher vaccination preferences and rates in older persons. From a mean follow-up period of 15 days after first dose, authors found that vaccine effectiveness increased with increase in time and receipt of second dose. The researchers also found BNT162b2 vaccine effectiveness against symptomatic COVID-19 to be 57% after 14–20 days, and 66% after 21–27 days. Although vaccine effectiveness was high, follow-up period may have reflected a narrower window for assessing maximum vaccine effectiveness after second dose. Other limitations associated with this study include potential selection bias and risk of potential confounding bias. Exclusion of some eligible participants as a result of efforts to match may also have influenced study outcomes. Meanwhile, findings from the study were strengthened by the large study size which allowed estimation of vaccine effects on several outcomes. The use of a test negative case–control may have controlled some potential confounders.

In another Israeli retrospective study, 51.0% effectiveness was observed by Chodlick et al. (2021) during 24 days after first dose of BNT162b2 vaccine. The study involved 503, 875 cohort who received first dose of the vaccine between December 19 2020 and January 17 2021. Cumulative incidence of SARS-CoV-2 infection reduced from 0.57% (after 1–12 days of first dose) to 0.27% (after 13–24 days of first dose).This is suggestive of increase in effectiveness with increase in time, and the high relevance of a second dose, for enhanced effectiveness of the vaccine in preventing COVID-19. The study included persons of ≥ 16 years of age and findings were similar across gender, age and communities. Potential unreported vaccination among potential eligible participants may account for a limitation in this study. This study was yet to be peer-reviewed as at the time of this review.

Researchers from England studied the effectiveness of BNT162b2 and ChAdOx1 vaccines against confirmed COVID-19; particularly for the UK variant, associated hospitalisation and death (Bernal et al. 2021a). The study included a cohort of over 70 year olds from December 8 2020 to February 19 2021. For participants aged 80 years old and above, vaccine effectiveness was observed 10–13 days after first dose, and reached an effectiveness of 70%. It was observed that vaccination with either a single dose of BNT162b2 or ChAdOx1 vaccines was associated with significant reduction in symptomatic COVID-19 cases in older persons. The vaccines were also associated with enhanced protection from disease severity among recipients. Introduction of a second dose of BNT162b2 provided increased protection from symptomatic disease. There was insufficient follow-up of persons who received second dose of ChAdOx1 vaccine, to fully assess its effectiveness after second dose. However, both vaccines were estimated to have equivalent effectiveness. Single dose of either vaccine was estimated to be 80.0% at preventing hospitalisation, and single dose of BNT162b2 was 85.0% effective at preventing COVID-19-related death. ChAdOx1 vaccine effects were seen from 14 to 20 days after vaccination and reached effectiveness of 60.0% from 28 to 34 days and increased to 73.0% from 35 days onwards. Risk of emergency hospital admission and death was reduced by 43% and 51% respectively, by a dose of BNT162b2 as reported by the researchers. Meanwhile a dose of ChAdOx1 vaccine was associated with reduced COVID-19-related emergency by 37%. Confounders such as age, gender, ethnicity and geographic region were included in the logistic regression model. The retrospective study was performed using 174,731 pillar 2 PCR tested samples which included study and control groups. Findings from this study suggest faster onset of observable effectiveness with BNT162b2 than with ChAdOx1 vaccine in older persons, however the study was not yet peer-reviewed as at the time of this review.

A retrospective study by Helms-Mousten et al. (2021) in Denmark assured of BNT162b2 effectiveness within and after 7 days of the second dose in the general population. The study included 39,040 persons of 77–90 years old, and 33,039 persons of 36–57 years old. A total of 95.2% and 86. 0% of the older persons received first and second dose of the vaccine respectively, from 27 December 2020 to 18 February 2021, while 27.8% and 24.4% of younger group received the vaccine doses respectively. No protective effect was observed in the older group after first dose of vaccine, while vaccine effectiveness of 17% was seen in the younger group after 14 days of first dose. Furthermore, vaccine effectiveness of 52% was seen in the older group after second vaccine dose (0–7 day after) and 46% in the younger group. Beyond 7 days of the second dose, vaccine effectiveness increased to 64% and 90% in the 2 groups respectively. This observation study shows a higher effectiveness of BNT162b2 vaccine in the younger population than in older persons after 7 days of second vaccine dose (full vaccination). This may suggest exploring the need for additional dose in older persons, however the study was yet to be peer-reviewed as at the time of the study, and findings were not validated yet. The observed variation in vaccine effectiveness in older and younger groups may have been influenced by the large variation in the sample size of the groups and potential confounders. However, more objective evidence is anticipated as more data emerge.

In a prospective cohort study by Vasileious et al. (2021) outcomes of first dose of BNT162b2 in Scotland was observed, and the vaccine was associated with significant reductions of risk of COVID-19-related hospital admissions. Evaluation of vaccine effectiveness was done using available vaccination data, primary care PCR testing and hospital admission records of 5.4 million persons in Scotland (99% of the population) registered at 940 general practices. Among a total of 1,331,993 vaccinated persons with mean age of 65 years old, first dose of BNT162b2 vaccine was associated with 91% reduced COVID-19-related hospitalisation at 28–34 days after vaccination. Effects of ChAdOx1 at same time interval was 88%. However, persons of 80 years old and above showed similar effects of BNT162b2 and ChAdOx1 against COVID-19-related hospitalisation. Vasileiou et al. have provided real-world evidence of equivalent effectiveness of BNT162b2 and ChAdOx1 COVID-19 vaccines. As common with observational studies, the risk of potential inherent confounder bias may not be exempted in the study.

Similarly, in studying the effectiveness of BNT162b2 and ChAdOx1 COVID-19 vaccines on COVID-19 related mortality, Bernal et al. (2021b) associated 44% and 69% reduced risk of death with one and two respective doses of BNT162b2 vaccination among COVID-19 cases. Similarly, a single dose of ChAdOx1 was described to be associated with 55% reduced risk of death from COVID-19. The study included a total of 48,096 vaccinated and unvaccinated older persons of ≥ 70 years old who were confirmed COVID-19 cases in England. The case–control study included retrospective data from December 8, 2020 to April 6, 2021, and hazard ratios for death was estimated within 28 days of positive SARS-CoV-2 PCR test by vaccination status. Due to the observational nature of the study, influence of potential unmeasured or residual confounders may not be completely excluded. Other causes of death within 28 days after vaccination were not investigated, and death may not be related to COVID-19 as suggested in the study. The study was also yet to be validated by peer-review prior to this review. However, findings provides evidence of associated high protection against mortality after a dose of BNT162b2 and ChAdOx1 COVID-19 vaccines.

In another study Shrotri et al. (2021) observed a decline in PCR positive tests to SARS-CoV-2 infection after vaccination with either BNT162b2 or ChAdOx1 in older persons in long-term care facilities (LTCF) in England. A retrospective data of 10,412 residents at 310 LTCF, from December 8, 2020 to March 15, 2021 were studied, and the researchers noted that a single dose of BNT162b2 or ChAdOx1 was significantly associated with reduced risk of SARS-CoV-2 infection in this category of persons. From the Adjusted hazard ratio, the study showed a decline to 0.44 and 0.38 at 28–34 days and 35–48 days respectively, post single dose of vaccine. Observed effects were similar between BNT162b2 (0.32) vaccine and ChAdOx1 (0.35) vaccine at days 35–48 post vaccination. The study elicits evidence of first dose-related reduction of SARS-CoV-2 infection in a care home, however the study was yet to be peer-reviewed as at the time of this review. Risk of potential confounder bias may also be associated with the study.

Hall et al. (2021), prospectively understudied a cohort of healthcare staff across multicentre in England between December 8, 2020 and February 5, 2021. Researchers documented all baseline risk factors and symptoms every other week, including all SARS-CoV-2 PCR tests and antibody test results. Hazard ratio was employed to compare time to infection in vaccinated and unvaccinated study participants to estimate the impact of BNT162b2 vaccine. It was noted in the study that BNT162b2 was associated with 72% effectiveness after 21 days of first dose, and 86% effectiveness seven days after second dose. This study demonstrates the effectiveness of BNT162b2 in preventing symptomatic and asymptomatic SARS-CoV-2 infection. It also demonstrates a potential effectiveness against the B1.1.7 variant which was predominant as at the time of the study.

Also, Pritchard et al. (2021) used a large community-based survey of individuals living in randomly selected private households across the United Kingdom, to assess the effectiveness of the BNT162b2 and ChAdOx1 nCoV-19 vaccines against any new SARS-CoV-2 PCR-positive tests. This was split according to self-reported symptoms, cycle threshold value (< 30 vs. ≥ 30; as a surrogate for viral load) and gene positivity pattern (compatible with B.1.1.7 or not). Using 1,945,071 real-time PCR results from nose and throat swabs taken from 383,812 participants between December 1, 2020 and May 8, 2021, the researchers found that vaccination with the ChAdOx1 or BNT162b2 vaccines already reduced SARS-CoV-2 infections ≥ 21 days after the first dose at 61% and 66% respectively. Higher reductions was also observed after a second dose of each vaccine at 79% and 80% respectively. According to the study, the largest reductions were observed for symptomatic infections and/or infections with a higher viral burden. Overall, COVID-19 vaccination reduced the number of new SARS-CoV-2 infections, with the largest benefit received after two vaccinations. Effectiveness was noted against symptomatic and high viral burden of infections, and no evidence of a difference was observed between BNT162b2 and ChAdOx1 vaccines. Through their comparison, Pritchard et al. demonstrated similar effectiveness between BNT162b2 and ChAdOx1 vaccines.

### Effectiveness of BNT162b2 vaccine and ChAdOx1 COVID-19 vaccines against variants of SARS-CoV-2

In an Israeli national study, Haas et al. (2021) observed that two doses of BNT162b2 are highly effective in all ages (16 years to ≥ 80 years), against B.1.1.7 variant, symptomatic and asymptomatic SARS-CoV-2 infections including, COVID-19 related hospitalisation and death. According to the authors, increase in vaccine coverage had a corresponding marked and sustained decline in SARS-CoV-2 incidence. Retrospective data from January 24 to April 3, 2021 were analysed to compare rates of SARS-CoV-2 outcomes in fully vaccinated and unvaccinated cohorts. At 7 days after second dose of BNT162b2, 95.3% effectiveness against SARS-CoV-2 incidence was reported in the study, while 91.5% effectiveness against asymptomatic SARS-CoV-2 infection, 97.0% against symptomatic COVID-19, 97.2% against COVID-19 related hospitalisation, and 96.7% against COVID-19 related deaths were noted. While providing evidence that two doses of BNT162b2 vaccines are associated with high effectiveness, the potential influence of confounders is noteworthy.

Again, Bernal et al. (2021c) compared the effectiveness of BNT162b2 vaccine and ChAdOx1 vaccine against B.1.6172.2 and B.1.1.7 variants of SARS-CoV-2. There was significantly lower effectiveness of one dose of vaccine against the B.1.617.2 than B.1.1.7 variant (33.5% and 51.1% respectively), as reported by the researchers. Therefore, absolute reduction of one dose vaccine effectiveness against B.1.6172.2 variant was approximately 20%, when compared to effectiveness against B.1.1.7 variant. More specifically, dose effectiveness of BNT162b2 reduced from 93.4% with B.1.1.7 to 87.9% with B.1.6172.2. Meanwhile, the ChAdOx1 vaccine associated effectiveness of two doses reduced to 66.1% with B.1.1.7 to 59.8% with B.1.6172.2. The researchers therefore concluded that only a modest difference in vaccine effectiveness against B.1.6172.2 and B.1.1.7 variants of SARS-CoV-2 was associated with two doses of either vaccine. The retrospective study included vaccination data up to May 16, 2021, on date of each vaccine dose and type of vaccine administered. The study showed significant effectiveness against symptomatic COVID-19 after two vaccine doses, and supports the current use of two doses, particularly in the face of B.1.6172.2 variant. Potential misclassification of cases and control may have been influenced by sensitivity and specificity of the PCR. Potential confounding resulting from difference in vaccine coverage in population groups may also be associated with the study. Additionally, this paper was yet to be peer-reviewed as at the time of this review, therefore its findings and study methods were yet to be validated.

## Discussion

Although data availability was limited, the studies suggest equivalent effectiveness of BNT162b2 and ChAdOx1 COVID-19 vaccine against SARS-CoV-2 infection and COVID-19 related morbidity and mortality. However, a modest difference was noted by one study (Bernal et al. 2021a) in the effectiveness of the two vaccines against B.1.6172.2 and B.1.1.7 variants of SARS-CoV-2. More data is required for improved understanding and more objective conclusions about this finding. Meanwhile, effectiveness of the vaccines generally appeared to vary with the number of days in different locations, particularly in older persons, nonetheless the vaccines showed effectiveness in younger and older persons. This is similar to results of interim analysis of BNT162b2 which indicated efficacy against COVID-19 across all ages (Polack et al. 2020). More data from the general population is required for enhanced evidence and clarity on variations in time interval prior onset of effectiveness among different populations. As studies continue to emerge, it is hoped that more evidence will be made available.

It was also noted that vaccine effectiveness after first dose increased with increase in time. Effectiveness was mostly seen after 7 days of first dose, and this appears to further increase to about 90% after 28 days of first dose. In congruence, Amit et al. in their correspondence suggested the possibility of occurrence of COVID-19 symptoms within a median time of 3.5 days post vaccination (Amit et al. 2021a). This reinforces the need for continued use of other precautionary measures even after vaccination, particularly within the first few days of post vaccination. Similarly, in a reanalysis of a previous work, Hunter and Brainard (2021) also noted and associated high effectiveness of single dose of BNT162b2 to about 21 days post vaccination. In a correspondence by Sheikh et al. (2021), vaccine effectiveness against SARS-CoV-2 delta variant of concern, did not clearly manifest until at least 28 days post vaccination with a single dose. In their study, McDonald et al. observed that optimal immune response to BNT162b2 is achieved by a boost dose, particularly in older persons (McDonald et al. 2021), reinforcing the need for a second dose.

Both vaccines were associated with preventing incidence of SARS-CoV-2 infection, COVID-19 related hospitalisation and death. In a correspondence, Amit et al. associated rate reduction of SARS-CoV-2 infection to 30% and 75% for days 1–14 and 15–28, respectively after first dose of BNT162b2 vaccine (Amit et al. 2021b). Meanwhile adjusted rate reduction of COVID-19 was associated with 47% and 85% for days 1–14 and 15–28 respectively, after first dose of BNT162b2 (Amit et al. 2021b). This finding agrees with the current practice of delaying the second dose, to ensure wider coverage and protection, particularly in places with vaccine shortage. Future directions of research points towards identifying the duration of effectiveness of the vaccines after a second dose. This will enhance strategic vaccination and improved public health. A focus on vaccine effectiveness across demographics such as gender, age groups and ethnicity, will also provide enhanced knowledge of available COVID-19 vaccines.

This review has provided a systematic evidence of high effectiveness of BNT162b2 mRNA vaccines and ChAdOx1 adenovirus vaccine against SARS-CoV-2 infection and COVID-19, and encourages wider coverage to facilitate pandemic control. However, a large majority of the studies were yet to be validated by peer-review, and as such are not recommended for clinical decisions. In addition, all the studies were observational and so had inherent potential risk of bias associated with observational studies. Potential risk of confounder bias may have also influenced findings in the studies. The review process may have also introduced some limitations to this study, among which may include potential selection bias. Similarly, the inclusion of heterogeneous studies with varying methodologies; nationwide cohorts, Case–control, test negative case–control and Screening methods, may have introduced some bias following varying methods of evaluating vaccine effectiveness. Again, more studies on BNT162b2 vaccine, than ChAdOx1 vaccine were available, and this may have resulted in some level of bias in the conclusions.

## Conclusions

BNT162b2 mRNA vaccine and ChAdOx1 adenovirus vaccine were observed to be associated with equivalent and high effectiveness against SARS-CoV-2 infection and COVID-19 related morbidity and mortality in the general population. Vaccine effectiveness were observed to be mostly seen after 7 days of initial dose, and increased steadily to about 35 days, with an enhanced effectiveness following the second dose. This supports and encourages the continued practice of other preventive measures, particularly during the first week of vaccination, while also reinforcing the need for a second vaccine dose. Increase in single dose vaccine effectiveness over times supports the initiative of a delayed second dose to maximise benefit.

## Abbreviations

SARS-CoV-2: Severe acute respiratory syndrome coronavirus 2
COVID-19: Coronavirus disease 2019
mRNA: messenger Ribonucleic Acid
ChAdOx1: Chimpanzee Adenovirus-vector vaccine by Oxford
BNT: BioNTech

## References

[CR1] Amit S, Beni SA, Biber A, Grinberg A, Lashem E, Regev-Yochay G (2021). Post vaccination COVID-19 among healthcare workers, Israel. Emerg Infect Dis.

[CR2] Amit S, Regev-Yochay G, Afek A, Kreiss Y, Leshem E (2021). Early rate reduction of SARSCoV-2 infection and COVID-19 in BNT162b2 vaccine recipients. Lancet.

[CR4] Bernal JL, Andrew N, Gower C, Stowe J, Robertson C, Tessier E, Simmons R, Cottrell S, Roberts R, O’Doherty M, Brown K, Cameron C, Stockton D, McMenamin J, Ramsay M (2021). Early effectiveness of Covid-19 vaccination with BNT162b2 mRNA vaccine and ChAdOx1 adenovirus vector vaccine on symptomatic disease, hospitalization and mortality in older adults in England. MedRxiv.

[CR5] Bernal JL, Andrews N, Gower C, Gallagher E, Simmons R, Thelwall SJ (2021). Effectiveness of COVID-19 vaccines against B.1.617.2 variant. MedRxiv.

[CR3] Bernal JL, Andrews N, Gower C, Stower J, Tessier E, Simmons R, Ramsay M (2021). Effectiveness of BNT162b2 mRNA vaccine and ChAdOx1 adenovirus vector vaccine on mortality following COVID-19. MedRxiv.

[CR6] Chodlick G, Tene L, Patalon T, Gazit S, Tov AB, Cohen D, Muhsen K (2021). The effectiveness of the first dose of BNT162b2 vaccine at reducing SARS.CoV-2 infection 13–24 days after immunization: real world evidence. MedRxiv.

[CR7] Cooper DM, Afghani B, Byington CL, Cunningham CK, Golub S, Lu KD, Radom-Aizik S, Ross LF, Singh J, Smoyer WE, Lucas CT, Tunney J, Zaldivar F, Ulloa ER (2021). SARS-CoV-2 vaccine testing and trials in the paediatric population: biologic, ethical, research, and implementation challenges. Pediatric Res.

[CR8] Dagan N, Barda N, Kepten E, Miron O, Perchik S, Kartz MA, Hernan MA, Lipsitch M, Reis B, Balicer RD (2021). BNT162b2 mRNA Covid-119 vaccine in a nationwide mass vaccination setting. N Engl J Med.

[CR9] Department of health and social care. Statement from the UK Chief Medical Officers on the prioritisation of first doses of COVID-19 vaccines. https://www.gov.uk/government/news/statement-from-the-uk-chief-medical-officers-on-the-prioritisation-of-first-doses-of-covid-19-vaccines.

[CR10] Forni G, Mantovani A (2021). On behalf of COVID-19 commision of Academia Nazionale dei Lincei, Rome. Cell Death Differ.

[CR11] Haas EJ, Angulo F, McLaughlin JM, Anis E, Singer SR, Khan F, Brooks N, Smaja M, Mircus G, Pan K, Southern J, Swerdlow DL, Jorder L, Levy Y, Alry-Preis S (2021). Impact and effectiveness of mRNA, BNT162b2 vaccine against SARS-CoV-2 infections and COVID-19 cases, hospitalisations, and deaths following a nationwide vaccination campaign in Israel: an observational study using national surveillance data. Lancet.

[CR12] Hall VJ, Foulkes S, Saei A, Andrews N, Oguti B, Charlett A (2021). COVID-19 vaccine coverage in health-care workers in England and effectiveness of BNT162b2 mRNA vaccine against infection (SIREN): a prospective, multicentre, cohort study. The Lancet.

[CR13] Helfand BKI (2020). The exclusion of older persons from vaccine and treatment trials for COVID-19. J Am Med Assoc.

[CR14] Helms-Mousten IR, Emborg H, Nielsen J, Neilsen KF, Krause TG, Molbak K, Moller KL, Berthelsen AN, Valentiner-Branth P (2021). Vaccine effectiveness after first and second dose of the BNT162b2 mRNA COVID-19 vaccine in longterm care facility residents and healthcare workers: a Danish cohort study. MedRxiv.

[CR15] Hunter PR, Brainard J (2021). Estimating effectiveness of the Pfizer COVID-19 BNT162b2 vaccine after a single dose (2021) A reanalysis of a study of “real world” vaccination outcomes from Israel. MedRxiv.

[CR16] McDonald I, Murray SM, Reynolds CJ, Altmann DM, Boyton RJ (2021). Comparative systematic review and meta-analysis of reactogenicity, immunogenicity and efficacy of vaccines against SARS-CoV-2. NPJ Vac.

[CR17] Moher D (2009). Preferred reporting items for systematic reviews and meta-analysis: The PRISMA statement. PLoS Med.

[CR18] National Heart, Lung, and Blood Institute. Study Quality Assessment Tools. https://www.nhlbi.nih.gov/. Accessed May 31, 2021.

[CR19] Nicola M, Alsafi Z, Sohrabi C, Kerwan A, Al-Jabir A, Iosifidis C, Agha M, Agha R (2020). The socio-economic implications of the coronavirus pandemic (COVID-19): a review. Int J Surg.

[CR20] Polack FP, Thomas SJ, Kitchen N, Absalon J, Gurtman A, Lockhart S, Perez JL (2020). Safety and efficacy of the BNT162b2 mRNA COVID-19 vaccine. N Engl J Med.

[CR21] Pritchard E (2021). Impact of vaccination on new SARS-CoV-2 infections in the United Kingdom. Nat Med.

[CR22] Raj N, Fernandes S, Charyulu NR, Dubey A, Ravi GS, Hebbar S (2019). Postmarket surveillance: a review on key aspects and measures on the effective functioning in the context of the United Kingdom and Canada. Ther Adv Drug Saf.

[CR23] Sanyaolu A, Okorie C, Hossein Z, Patidar R, Desai P, Prakash S, Jaferi U, Mangat J, Marinkovic A (2020). Global pandemicity of COVID-19: situation report as of June 9, 2020. Infect Dis Res Treat.

[CR24] Sheikh A, McMenamin J, Taylor RC (2021). SARS-CoV-2 Delta VOC in Scotland: demographics, risk of hospital admissions, and vaccine effectiveness. Lancet.

[CR25] Shrotri M, Krutikov M, Palmer T, Giddings R, Azmi B, Subbarao S, Fuller C, Irwin-Singer A, Davies D, Tut G, Bernal JL, Moss P, Hayward A, Copas A, Shallcross L (2021). Vaccine effectiveness of the first dose of ChAdOx1 nCoV-19 and BNT162b2 against SARS-CoV-2 infection in residents of long-term care facilities (VIVALDI study). MedRxiv.

[CR26] Vasileious E, Simpson CR, Shi T, Kerr S, Agrawal U, Akbari A, Bedston S, Beggs J, Bradly D, Chuter A, de Lusignan S, Marple J, McCowan C, McGagh D, McMenamin J, Moore E, Murray JL, Pan J, Ritchie L, Shah SA, Stock S, Torabi F, Tsang RS, Wood R, Woolhouse M, Robertson C, Sheikh A (2021). Interim findings from first dose mass COVID-19 vaccination roll-out and COVID-19 hospital admission in Scotland: a national prospective cohort study. Lancet.

[CR27] Voysey M, Clemens SAC, Madhi SA, Weckx LY, Folegatti PM, Aley PK (2021). Safety and efficacy of the ChAdOx1 nCoV-19 vaccine (AZD1222) against SARS-CoV-2: an interim analysis of four randomised controlled trials in Brazil, South Africa, and the UK. Lancet.

[CR28] Welch MJ, Lally R, Miller JE, Pittman S, Brodsky L, Caplan AL, Uhlenbrauck G, Louzao DM, Fischer JH, Wilfond B (2015). The ethics and regulatory landscape of including vulnerable populations in pragmatic clinical trials. Clin Trials.

[CR29] World Health Oganisation (WHO) WHO issues its first emergency validation for a COVID-19 vaccine and emphasizes need for equitable global service. https://www.who.int/home/news/. Accessed June 19, 2021a.

[CR30] World Health Organisation. https://covid-19.who.int/info. Accessed 19 June 19 2021b.

